# Making progress towards food security: evidence from an intervention in three rural districts of Rwanda

**DOI:** 10.1017/S1368980015002207

**Published:** 2015-08-06

**Authors:** Vincent Nsabuwera, Bethany Hedt-Gauthier, Mohammed Khogali, Mary Edginton, Sven G Hinderaker, Marie Paul Nisingizwe, Jean de Dieu Tihabyona, Benoit Sikubwabo, Samuel Sembagare, Antoinette Habinshuti, Peter Drobac

**Affiliations:** 1 Partners In Health/Inshuti Mu Buzima, PO Box 3432, Kigali, Rwanda; 2 Harvard Medical School, Boston, MA, USA; 3 Médecins sans Frontières (MSF)/Luxembourg; 4 International Union Against Tuberculosis and Lung Diseases, Paris, France; 5 School of Public Health, University of the Witwatersrand, Johannesburg, South Africa; 6 Centre for International Health, University of Bergen, Norway; 7 Kirehe District, Ministry of Local Government, Rwanda; 8 Kayonza District, Ministry of Local Government, Rwanda; 9 Burera District, Ministry of Local Government, Rwanda; 10 Division of Global Health Equity, Brigham and Women’s Hospital, Boston, MA, USA

**Keywords:** Food security, Food accessibility, Food consumption, Operational research

## Abstract

**Objective:**

Determining interventions to address food insecurity and poverty, as well as setting targets to be achieved in a specific time period have been a persistent challenge for development practitioners and decision makers. The present study aimed to assess the changes in food access and consumption at the household level after one-year implementation of an integrated food security intervention in three rural districts of Rwanda.

**Design:**

A before-and-after intervention study comparing Household Food Insecurity Access Scale (HFIAS) scores and household Food Consumption Scores (FCS) at baseline and after one year of programme implementation.

**Setting:**

Three rural districts of Rwanda (Kayonza, Kirehe and Burera) where the Partners In Health Food Security and Livelihoods Program (FSLP) has been implemented since July 2013.

**Subjects:**

All 600 households enrolled in the FSLP were included in the study.

**Results:**

There were significant improvements (*P*<0·001) in HFIAS and FCS. The median decrease in HFIAS was 8 units (interquartile range (IQR) −13·0, −3·0) and the median increase for FCS was 4·5 units (IQR −6·0, 18·0). Severe food insecurity decreased from 78 % to 49 %, while acceptable food consumption improved from 48 % to 64 %. The change in HFIAS was significantly higher (*P*=0·019) for the poorest households.

**Conclusions:**

Our study demonstrated that an integrated programme, implemented in a setting of extreme poverty, was associated with considerable improvements towards household food security. Other government and non-government organizations’ projects should consider a similar holistic approach when designing structural interventions to address food insecurity and extreme poverty.

Food security is achieved ‘when all people, at all times, have physical, social and economic access to sufficient, safe, and nutritious food to meet their dietary needs and food preferences for an active and healthy life’^(^ ^1^ ^)^. Despite global efforts to achieve the Millennium Development Goal 1 of halving hunger by 2015, over 800 million people were still estimated to be chronically undernourished in 2014 with the highest prevalence in sub-Saharan Africa, where approximately one in four people are undernourished^(^ ^2^ ^)^.

Although Rwanda has made considerable socio-economic progress and poverty has been reduced by over 50 % in the two decades since the 1994 genocide^(^ ^3^ ^)^, challenges with household food insecurity and malnutrition remain. Over 43 % of childhood deaths in the country are attributed to malnutrition and 44 % of children under the age of 5 years are stunted^(^ ^4^ ^)^. The major cause of children’s chronic malnutrition in Rwanda is the inadequate quantity and quality of food consumed at the household level^(^ ^5^ ^)^. Seasonal difficulties in accessing adequate food persist for 51 % of households, with 14 % having constant problems all year round^(^ ^6^ ^)^.

Food insecurity and malnutrition are complex problems that need to be tackled in a coordinated way with political commitment and leadership^(^ ^2^ ^)^. In this regard and in line with the 2011 Joint National Action Plan to Fight Malnutrition in Rwanda, Partners In Health (PIH) – a US-based non-governmental health organization operating in Rwanda since 2005 – launched in 2013 the Food Security and Livelihoods Program (FSLP) in three rural districts of Rwanda. The immediate goal of this initiative was to increase food accessibility and consumption for extremely poor and vulnerable households, with the long-term aim of reducing malnutrition and improving health. The multifaceted intervention package included a one-time capital investment including agricultural inputs support, assistance with small livestock projects, provision of microloans and nutrition education (Box 1).

Box 1 Partners In Health/Inshuti Mu Buzima (PIH/IMB) Food Security and Livelihoods Program in Rwanda: intervention components

**Table:** 

Key component	Details
Promoting group work	∙ Participating households are encouraged to form small self-help groups (15–20 households based on proximity), with a goal of creating formal business cooperatives
	∙ The groups manage access to small loans and rent additional land for farming businesses
Trainings and support to increase the crop	∙ Composting and fertilizer use
yields on household owned small land plots	∙ Ideal selection and rotation of crops, emphasizing vegetable production for home consumption and market
	∙ Identifying and using quality seeds, with a focus on nutritional value
	∙ Using best techniques of planting including seed application and spacing
	∙ Pest and diseases management
Land renting and horticulture business promotion	∙ Programme participants are encouraged to rent additional land (as a group) and focus on growing high-yielding crops for income generation
Small livestock rearing	∙ Participating households are encouraged and trained on poultry rearing (chickens and rabbits) as well as other easy-to-raise animals like pigs, sheep or goats
Promoting voluntary savings and group loans	∙ Each member of the self-help groups is encouraged to come in the meeting with a small amount of savings every week, which creates a group treasury for loans
Training and mentorship on business planning and management	∙ Ongoing trainings and follow-up coaching are offered to the self-help groups to support innovative income-generating ideas
Access to microloans for expansion of farm business and off-farm income generation	∙ Programme participants within their groups apply for small (individual or group) loans with a low interest rate during the first year with the microloans package of Partners In Health, while transitioning to community-based microfinance institutions and banks
Nutritional knowledge transfer	∙ Trainings on basics of nutrition and diet preparation
	∙ Community cooking demonstrations
	∙ Tailored trainings and mentorship for pregnant women, including diet, breast-feeding and child complementary feeding
	∙ Household hygiene and sanitation

In the present study we aimed to measure the impact of the FSLP on the Household Food Insecurity Access Scale (HFIAS) score (Box 2) score and household Food Consumption Score (FCS; Box 3) to assess whether food security had improved during a 12-month period. Specific objectives of the study were to: (i) describe baseline demographic and socio-economic characteristics of the 600 households selected in three rural districts; (ii) assess changes in the HFIAS and FCS after a one-year implementation of integrated food security interventions; and (iii) identify associations between selected socio-economic factors and the changes in HFIAS and FCS.

Box 2 Household Food Insecurity Access Scale (HFIAS) score as a measurement of food access

The HFIAS is a nine-question tool developed and validated by the Food and Nutrition Technical Assistance to assess household food insecurity, and looks at three key domains experienced in households during the previous month:

1. Stated anxiety and uncertainty about food.
2. Household experience with quality of food (variety and preferences).
3. Insufficient household food intake (quantity).

The nine questions are as follows, referring to the past 30 d:

Q1. Did you worry that your household would not have enough food?
Q2. Were you or any household member not able to eat the kinds of food you preferred because of lack of resources?
Q3. Did you or any household member eat just a few kinds of food day after day due to lack of resources?
Q4. Did you or any household member eat food that you preferred not to eat because of lack of resources to obtain other types of food?
Q5. Did you or any household member eat a smaller meal than you felt you needed because there was not enough food?
Q6. Did you or any household member eat fewer meals in a day because there was not enough food?
Q7. Was there ever no food at all in your household because there were not enough resources to get more?
Q8. Did you or any household member go to sleep at night hungry because there was not enough food?
Q9. Did you or any household member go a whole day without eating anything because there was not enough food?

Each question’s score depends on how frequent the household has lived with that situation in the past 30 d: ‘never happened’=0; ‘rarely’ (once or twice)=1; ‘sometimes’ (three to ten times)=2; or ‘often’ (more than ten times)=3. A total score for the household ranges on a scale from 0 to 27. A higher HFIAS score is indicative of poorer access to food and greater household food insecurity.

Prevalence of food insecurity is further categorized as follows:

1. Food secure: if (Q1=0 or Q1=1) and all other questions=0.
2. Mildly food insecure: if (Q1=2 or Q1=3 or Q2=1 or Q2=2 or Q2=3 or Q3=1 or Q4=1) and (Q5, Q6, Q7, Q8, Q9=0).
3. Moderately food insecure: if (Q3=2 or Q3=3 or Q4a=2, Q4=3 or Q5=1 or Q5=2 or Q6=1 or Q6=2) and (Q7=0 and Q8=0 and Q9=0).
4. Severely food insecure: if (Q5=3 or Q6=3 or Q7=1 or Q7=2 or Q7=3 or Q8=1 or Q8=2 or Q8=3 or Q9=1 or Q9=2 or Q9=3).

Box 3 Food Consumption Score (FCS) measurement tool

FCS is a composite score based on varieties of food consumed by a household during the week before an interview (developed and validated by the World Food Programme):

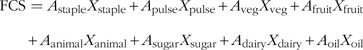

, where *X*_*i*_ is the frequency of food consumption (=number of days on which food group *i* was consumed during the past 7 d) and *A*_*i*_ is the weight of food group *i*.

The following weights were validated to be applied to food groups based on the energy, protein and micronutrient densities of each: meat, milk and fish=4; pulses=3; staples=2; vegetables and fruits=1; and sugar and oil=0·5.

Aggregated scores are categorized as follows:

1. poor food consumption (FCS=0–21);
2. borderline food consumption (FCS=21·5–35); and
3. acceptable food consumption (FCS>35).

## Methods

### Study design

This is a before-and-after intervention study, comparing food access and consumption scores at baseline and following the 12-month implementation of an integrated food security intervention.

### General setting

Rwanda is a small land-locked country in East Africa, with a population of approximately 10·7 million people^(^ ^7^ ^)^. The country has limited natural resources and the highest population density in continental Africa^(^ ^8^ ^)^. Agriculture is the backbone of Rwanda’s economy, accounting for 80 % of the labour force, with 90 % of these being subsistence farmers^(^ ^9^ ^)^.

### Study sites and population

The FSLP was implemented in three rural districts of Rwanda: Kayonza and Kirehe Districts in the Eastern Province and Burera District in the Northern Province. These are among the most remote and poorest districts in the country^(^ ^9^ ^)^. In consultation with district leadership, one administrative sector was selected for the intervention in each of the three districts, based primarily on the high rates of malnutrition and poverty and absence of development support from elsewhere. Within each sector, 200 households were identified through community participation and selected for the intervention. Specific criteria of selection included extreme poverty as defined nationally^(^ ^10^ ^)^, positive malnutrition screening for at least one member of the household and/or vulnerability to extreme poverty due to chronic diseases (such as HIV/AIDS, cancer or diabetes). All 600 households enrolled in the FSLP in July 2013 were included in the present study.

### Programme description

In designing the integrated programme of food security and livelihoods, PIH considered four interlinked pillars emanating from the food security concept^(^ ^11^ ^)^: (i) promoting food availability by increasing participants’ crop yields; (ii) food accessibility through diversifying sources of household income; (iii) food use and utilization through imparting tailored nutritional knowledge to participants by formal trainings and informal mentorship in home visits; and finally (iv) ensuring stability or sustainable access to food by promoting long-term cooperative enterprises. This holistic package of interventions included a one-time financial start-up support of 70 000 FRW ($US 100) to each participating household. This total was subdivided into 50 000 FRW ($US 70) to purchase agricultural products such as seeds, fertilizers and basic tools, and 20 000 FRW ($US 30) to purchase small livestock. This initial financial support was coupled with technical capacity building through a series of trainings on best farming techniques, financial literacy, cooperative formation, small projects design, loan applications and nutritional trainings (Box 1). In order to strengthen long-term income-generating enterprises, PIH established a fund for microloans in which the FSLP participants had equal opportunity to borrow money at low interest of 5 % while preparing them in utilizing community-based commercial microfinance institutions and banks for subsequent loans.

To ensure that the programme interventions were anchored in community management mechanisms, the programme used a peer-to-peer extension model. Exemplary lead farmers in the community were trained as trainers on agricultural techniques through farmer field schools. These trained agriculture assistant workers and the established structure of community health workers served as mentors for the participating households.

The FSLP was designed as a three-year intervention, with the first year dedicated to raising awareness of participants on their potential and available opportunities to become self-reliant, identifying and training the agriculture assistant workers and supporting programme participants with the agricultural interventions for two consecutive seasons. Transitioning to an entrepreneurship phase, the second year focuses on trainings in small-scale project design, business development and cooperative formation. The third year is focused on sustainability, with mentorship on cooperative management, and trainings on financial capital acquisition through loans and linking with microfinance institutions and banks. The current paper evaluates interim outcomes at the end of the first 12-month period.

### Data variables, sources and collection

Two internationally developed measurement tools, the Household Food Insecurity Access Scale (HFIAS) and the Food Consumption Score (FCS), were used to evaluate the programme (Boxes 2 and 3). Both have been validated internationally^(^ ^12^ ^,^ ^13^ ^)^.

The present study included data collected from the 600 households participating in the FSLP. The baseline was conducted in August 2013 and the annual evaluation was carried out in August 2014 after 12 months of enrolment in the intervention. Surveys were completed based on interviews with the head or senior member of each household. Demographic and socio-economic characteristics for the households recorded at baseline included household size, household dependency ratio (number of dependant members per number of active household members capable of work), household head marital status, land ownership, daily household income and overall household food insecurity prevalence. From the survey data, HFIAS and FCS were calculated at baseline and 12 months, using modular guidelines from the Food and Nutrition Technical Assistance^(^ ^14^ ^)^ and the World Food Programme^(^ ^15^ ^)^, respectively. The interviews were conducted during the same periods of the year (the dry summer season, August 2013 and August 2014).

To avoid potential bias and conflicts of interest of programme staff in the monitoring and evaluation process, for both baseline and end year evaluation, field interviews were conducted by an independent group of twelve young graduates hired temporarily and trained by the PIH Monitoring and Evaluation Department. Eight of the twelve data collectors were the same for both time points. Further, the Monitoring and Evaluation Department, which operates independently of any programme, also manages the databases, analyses and shares the results to respective programmes to keep track records of progress.

### Analysis and statistics

Data on variables of interest were exported for analysis from a Microsoft Access database to Epidata software version 2·2·2·182 (EpiData Association, Odense, Denmark). Sociodemographic characteristics were reported with proportions overall and by site. Depending on household response and level of frequency to each of the nine questions asked for HFIAS tabulation (Box 2), we followed an algorithmic method^(^ ^14^ ^)^ to categorize households into food secure, mildly food insecure, moderately food insecure and severely food insecure. FCS was categorized as poor (FCS=0–21), borderline (FCS=21·5–35) and acceptable (FCS>35) with respect to the World Food Programme’s technical guidance on cut-off points^(^ ^15^ ^)^.

The difference between before and after scores for each household was calculated by subtracting the baseline score from the score after one year. For HFIAS, a negative value indicates improvement, while for FCS a positive value indicates improvement. We tested for a change in scores using a Wilcoxon signed-rank test. We assessed whether the change in scores was significantly different across key socio-economic variables (geographic site, household size, household dependency ratio, household head marital status, land ownership and daily household income) using the Wilcoxon rank-sum test for categorical variables with two levels and the Kruskal–Wallis test for categorical variables with more than two levels. Levels of significance were set at 5 %.

### Ethics approval

Ethics approval was obtained from the Rwanda National Ethics Committee (RNEC). The study met also the criteria for studies of routinely collected data approved by the Médecins sans Frontières (MSF) Ethics Review Board (Geneva, Switzerland), and was approved by the Ethics Advisory Group of the International Union Against Tuberculosis and Lung Disease, Paris, France.

## Results

**Fig. 1:**
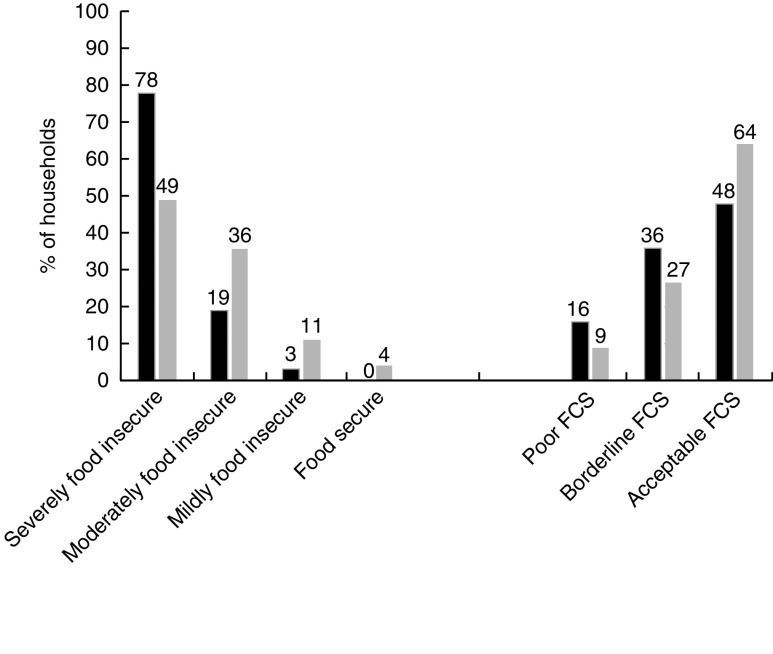
Proportion of household food insecurity and food consumption (FCS) levels at baseline (August 2013; 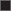) and one year after (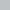) the food security programme intervention (August 2014) in three rural districts of Rwanda (FCS, Food Consumption Score)

From the 600 households enrolled in the FSLP, forty-six (7·7 %) did not have records of the one-year interviews and were classified as lost to follow-up. Table 1 shows baseline sociodemographic characteristics for the remaining 554 households. Ninety-four per cent were living on less than $US 1·50 daily and 89 % owned less than 0·2 ha of land. Most (78 %) had severe food insecurity. Over the course of one year, severe food insecurity decreased from 78 % to 49 %. FCS also improved after the intervention, with acceptable FCS increasing from 48 % to 64 % (Fig. 1).

**Table 1:** Baseline sociodemographic characteristics of the households included in the study in three rural districts of Rwanda, August 2013

	All districts		Kayonza		Kirehe		Burera	
Variable	*n*	%	*n*	%	*n*	%	*n*	%
Total	554	100	182	100	190	100	182	100
Household size								
<5 members	252	45	92	51	85	45	75	41
≥5 members	302	55	90	49	105	55	107	59
Household dependency ratio*								
≤1·5	340	61	99	54	121	63	120	66
>1·5	214	39	83	46	69	36	62	34
Household head marital status								
Husband and wife	499	90	178	98	160	84	161	89
Adult male, no wife	7	1	0	0	5	3	2	1
Adult female, no husband	47	9	4	2	25	13	18	10
Child headed (<18 years)	1	<1	0	0	0	0	1	<1
Household land ownership								
Land ≤2000 m^2^ (≤0·2 ha)	492	89	161	89	154	81	177	97
Land >2000 m^2^ (>0·2 ha)	62	11	21	12	36	19	5	3
Daily household income								
<1000 FRW ($US <1·5)	502	94	149	89	183	98	170	96
≥1000 FRW ($US ≥1·5)	30	6	19	11	4	2	7	4
Baseline food insecurity prevalence								
Mildly food insecure	14	2	1	<1	11	6	2	1
Moderately food insecure	105	19	27	15	45	24	33	18
Severely food insecure	427	78	151	83	129	68	147	81
Unknown	8	1	3	2	5	2	0	0

* The household dependency ratio is equal to (*n*_<5 years_+*n*_5–16 years_+*n*_65+ years_)/*n*_16–65 years_. 0=no dependants, 1=as many dependants as non-dependants, >1=more dependants than non-dependants.

Over one year, both HFIAS and FCS improved significantly (Table 2). The median change in HFIAS was 8 units (interquartile range (IQR) −13·0, −3·0; *P*<0·001). The median change in FCS was 4·5 units (IQR −6·0, 18·0; *P*<0·001). The improvement in HFIAS was significantly better for the poorest households with lower daily income (*P*=0·019) and the households with severe food insecurity at baseline (*P*<0·001). FCS improvement was significantly higher in households with bigger land compared with households with smaller land (*P*=0·03).

**Table 2:** Median changes in Household Food Insecurity Access Scale (HFIAS) score and household Food Consumption Score (FCS) after one year of programme intervention (2013/14) and associations with key sociodemographic characteristics in three rural districts of Rwanda

	Median change in HFIAS*			Median change in FCS*		
Sociodemographic variables	Median	IQR	*P* value	Median	IQR	*P* value
Overall	−8	−13·0,−3·0	<0·001†	4·5	−6·0, 18·0	<0·001†
Geographic site			0·09‡			0·89‡
1. Kayonza	−8	−12·0, −2·0		4·3	−6·0, 18·6	
2. Kirehe	−7	−12·0, −1·0		5·5	−10·3, 18·1	
3. Burera	−10·5	−15·0, −5·0		4·5	−3·0, 15·6	
Household size			0·56‡			0·24‡
<5 members	−8	−13·0, −3·0		6	−6·0, 19·0	
≥5 members	−8·5	−13·0, −3·0		3·5	−6·0, 16·5	
Household dependency ratio			0·45‡			0·99‡
≤1·5	−9	−13·0, −3·0		4·5	−6·0, 19·0	
>1·5	−7	−12·0, −3·0		4·7	−6·0, 16·0	
Household head marital status			0·89‡			0·41‡
Husband and wife	−8	−13·0, −3·0		4·5	−6·0, 18·0	
Adult male, no wife	−10	−19·0, −3·0		−2	−23·0, 16·0	
Adult female, no husband	−6	−12·0, −1·5		8	−2·5, 18·5	
Land ownership			0·15‡			0·03‡
Land ≤2000 m^2^ (≤0·2 ha)	−9	−13·0, −3·0		3·8	−6·0, 17·0	
Land >2000 m^2^ (>0·2 ha)	−5	−10·0, −0·0		9·7	−3·1, 19·6	
Daily household income			0·02‡			0·25‡
<1000 FRW ($US <1·5)	−8	−13·0, −3·0		4·8	−6·0, 18·0	
≥1000 FRW ($US ≥1·5)	−0·5	−8·0, 4·5		0·7	−8·1, 12·1	
Baseline food insecurity prevalence			<0·001‡			0·62‡
Mildly food insecure	0·5	−2·0, 3·0		3	−2·0, 18·0	
Moderately food insecure	−1	−4·0, 2·0		3	−7·5, 15·5	
Severely food insecure	−10	−14·0, −6·0		5	−6·0, 18·5	

IQR, interquartile range.

* Calculated by subtracting the baseline value from the endline value. For HFIAS, a negative score indicates improvement. For FCS, a positive score indicates improvement.

† Wilcoxon signed-rank test, testing whether the median change over time is significantly different from 0.

‡ *K*-sample test of equality of medians, assessing whether the change in scores is different between the groups.

## Discussion

The present study showed significant improvements in food accessibility and food consumption over a period of one year of FSLP implementation among the selected extremely poor households in three rural districts of Rwanda. Severe food insecurity levels decreased from 78 % to 49 % and acceptable food consumption improved from 48 % to 64 %. The impact was significantly greater for the poorest households.

A key priority of the Rwandan government over the past decade has been to reduce extreme poverty and malnutrition. The government has implemented a wide range of interventions to address these challenges, including the ‘one cow per family’ programme, the establishment of school gardens and the provision of subsidized fertilizers and free seeds to vulnerable families. The government has also promoted small livestock as a pathway to generate income for vulnerable households^(^ ^5^ ^)^. The financial support provided by the FSLP was comparable to similar government support to vulnerable families, such as the Vision 2020 Umurenge Program (VUP) that included direct cash transfers, averaging FRW 165 671 ($US 285) per household per year, and the provision of low-interest loans, averaging FRW 77 480 ($US 132) per household^(^ ^16^ ^)^. With these combined supports, extreme poverty across all ninety VUP sectors fell from 39·0 % in 2006/7 to 35·1 % in 2009^(^ ^16^ ^)^. The FSLP described here is aligned with those government initiatives and other non-governmental organizations have provided support within the same range. For example, the One Acre Fund, operating in Kenya and in seven districts of Rwanda, provides about $US 75 initial capital loan to farmers for seeds and fertilizers^(^ ^17^ ^)^.

Assessments of other integrated programmes addressing food security^(^ ^18^ ^,^ ^19^ ^)^ as well as community-based agricultural interventions^(^ ^20^ ^,^ ^21^ ^)^ have similarly demonstrated significant impact on one of the three pillars of food security (food availability; accessibility; use and utilization)^(^ ^11^ ^)^. However, these assessments have focused on single components of food security such as agriculture yield increases, household income, increases in vegetable consumption and diet diversification, and are not as comprehensive as our experience.

Recognizing the multidisciplinary and multidimensional aspects of food security, its measurement has been an ongoing challenge to researchers and practitioners^(^ ^22^ ^)^. Studies have revealed that segmented interventions such as community and home-based gardens, though positively correlated to food security, are unable to solve the problem of food insecurity^(^ ^23^ ^)^. It is advised that a long-term food security strategy incorporates not only agricultural food production interventions but also non-agricultural aspects to diversify livelihoods^(^ ^24^ ^,^ ^25^ ^)^. Also food security measurements should shift focus from distal proxy indicators to more fundamental measures, including subjective and psychological experiences of food insecurity^(^ ^26^ ^)^.

Common critiques of comprehensive interventions such as the FSLP are cost and sustainability^(^ ^27^ ^)^. The FSLP in our experience was an affordable community intervention tailored to the in-country social support programmes^(^ ^16^ ^)^. The one-time start-up investment of $US 100 per household, training costs and incentives to volunteer agriculture assistant workers in the form of transportation, lunch *per diems* and in-kind materials will be considered in future FSLP evaluations for the analysis of cost-effectiveness. A similar integrated food security programme in Malawi was found to be cost-effective with an average annual spending of $US 59 per household for a period of 6 years^(^ ^18^ ^)^.

Regarding programme sustainability, the second and third year of the FSLP are dedicated to emphasizing cooperative management, financial literacy and the utilization of microfinance institutions and banks. These aspects will facilitate the sustainability of the cooperatives as income-generating enterprises. Future assessments after 24 months and 36 months (years 2 and 3) will provide an opportunity to assess the maintenance of HFIAS and FCS improvements and, ultimately, the programme’s sustainability.

The present study has several strengths, including the use of a considerable sample size across three different agro-climatic zones of Rwanda. It assessed results of a programme that holistically targeted those most in need, who are often left out by macro-level development programmes. Internationally validated tools were used and STROBE (Strengthening the Reporting of Observational Studies in Epidemiology) guidelines^(^ ^28^ ^)^ were followed in the development of the current paper.

However, there are also some limitations to consider in the interpretation of the study. First, there was no control group and the results could be explained by temporal confounding. To our knowledge, during the course of the study year, there were no other development interventions that would have contributed to the registered significant changes apart from the FSLP in all the three sites. Changes in weather conditions could have affected food access; however, the Rwanda Agriculture Board recognized a reduction of agricultural production countrywide due to rain shortages^(^ ^29^ ^,^ ^30^ ^)^ corresponding to the same year considered in the present study. Had there been favourable weather conditions, the programme participants would likely have registered higher food access scores than those observed. The overall country socio-economic improvements could also have contributed to the change in living conditions of the population under study, although the short period seems to indicate that the major contributor was the FSLP.

Additionally, the HFIAS and FCS measurement tools have been criticized by some as being subjective^(^ ^31^ ^)^ or subject to response bias^(^ ^32^ ^)^, but the scores have been internationally validated as appropriate tools to assess food insecurity and strongly correlated with nutrition outcome^(^ ^33^ ^)^. To minimize respondents’ bias to HFIAS questions, these were the last part of a longer household assessment questionnaire inquiring about agriculture production, household income sources and household owned assets. Before data collection, interviewers were recommended to always probe any response in comparison to previous answers and actual observations. Further, we believe if there was a response bias, this bias would be consistent at baseline and follow-up, and would therefore reduce our ability to detect an improvement in HFIAS.

## Conclusion

The present study has shown potential for the FSLP to change the livelihoods of poor people in rural Rwanda. We believe there are several factors contributing to the success of this programme, including targeting of extremely poor households, using multifaceted and diversified small projects, promoting community participation and empowerment through self-help groups. Based on these findings, we recommend that other government and non-government organizations consider such integrated activities in the future, particularly when designing food security interventions aimed at uplifting the very poor.

While malnutrition, largely attributable to food insecurity, remains prevalent in sub-Saharan Africa and food and agricultural assistance programmes have been implemented broadly, there is little evidence demonstrating the impact of those programmes. The current paper provides such evidence of the potential impact. Given that achieving food security is a long-term endeavour, we will continue to assess the impact of the FSLP over several years to generate a set of benchmarks that could be recommended for similar programmes. Further, we recommend that future expansions of the programme be evaluated through a stepped-wedge randomized-controlled trial to truly isolate the impact FSLP on food consumption and security and other health outcomes.
